# Modeling violations of the race model inequality in bimodal paradigms: co-activation from decision and non-decision components

**DOI:** 10.3389/fnhum.2015.00119

**Published:** 2015-03-09

**Authors:** Michael Zehetleitner, Emil Ratko-Dehnert, Hermann J. Müller

**Affiliations:** ^1^Department Psychologie, Institut für Allgemeine und Experimentelle Psychologie, Ludwig-Maximilians-Universität MünchenMunich, Germany; ^2^Department of Psychological Sciences, Birkbeck College, University of LondonLondon, UK

**Keywords:** redundant signals effect, locus, co-activation, modeling, sequential sampling models, SRT, two-choice RT

## Abstract

The redundant-signals paradigm (RSP) is designed to investigate response behavior in perceptual tasks in which response-relevant targets are defined by either one or two features, or modalities. The common finding is that responses are speeded for redundantly compared to singly defined targets. This redundant-signals effect (RSE) can be accounted for by race models if the response times do not violate the race model inequality (RMI). When there are violations of the RMI, race models are effectively excluded as a viable account of the RSE. The common alternative is provided by co-activation accounts, which assume that redundant target signals are integrated at some processing stage. However, “co-activation” has mostly been only indirectly inferred and the accounts have only rarely been explicitly modeled; if they were modeled, the RSE has typically been assumed to have a decisional locus. Yet, there are also indications in the literature that the RSE might originate, at least in part, at a non-decisional or motor stage. In the present study, using a distribution analysis of sequential-sampling models (ex-Wald and Ratcliff Diffusion model), the locus of the RSE was investigated for two bimodal (audio-visual) detection tasks that strongly violated the RMI, indicative of substantial co-activation. Three model variants assuming different loci of the RSE were fitted to the quantile reaction time proportions: a decision, a non-decision, and a combined variant both to vincentized group as well as individual data. The results suggest that for the two bimodal detection tasks, co-activation has a shared decisional and non-decisional locus. These findings point to the possibility that the mechanisms underlying the RSE depend on the specifics (task, stimulus, conditions, etc.) of the experimental paradigm.

## Introduction

The human perceptual system consists of highly specialized sensory subsystems (for vision, audition, olfaction, etc.) which themselves are organized in a modular fashion. In order to adequately respond to the demands of a dynamically changing environment, the organism has to make countless decisions, which typically require the integration of signals from different modules—be it across modalities (multi-modal), within modalities (multi-feature), across different spatial locations (multi-location), or across different points in time.

Signal integration is frequently investigated using the so-called “redundant-signals paradigm” (RSP). For this paradigm, several statistical tools have been developed, which allow inferences to be drawn about the cognitive architecture and decisional mechanisms responsible for signal integration. In the RSP, participants are presented either with one of two possible single targets (e.g., a single auditory tone or a single visual flash) or with both targets redundantly (a tone and a flash). In general, the response times are, on average, faster for redundant-signal trials (RSTs) compared to single-signal trials (SSTs). This speed-up of response times, first reported by Todd (1912), is termed “redundant-signals effect” (RSE). It has since been replicated for a great variety of sensory modalities, tasks, and response categories as well as populations (see e.g., Grice et al., 1984; Diederich and Colonius, 1987; Mordkoff and Yantis, 1991; Krummenacher et al., 2002; Iacoboni and Zaidel, 2003; Miller and Reynolds, 2003; Gondan et al., 2004; Koene and Zhaoping, 2007; Schröter et al., 2007; Zehetleitner et al., 2009; Töllner et al., 2011; Krummenacher and Müller, 2014).

What type of processing architecture is underlying the RSE? The first architecture introduced to explain the RSE was the separate-activations or race model. Race models assume that the two stimulus properties of redundant targets are processed in parallel, in separate channels. According to this model, the shortening of response times for redundant relative to single targets derives from the fact that either target channel alone can trigger a response. As one of the two racers is stochastically faster than the other, the minimum time of both is, on average, shorter than that required by any racer alone. More formally, if one conceives of the triggering times of each channel as random variables, *X*_1_ and *X*_2_, on RSTs, the race can be expressed as the minimum of both variables. The expected value of this minimum is smaller than (or equal to) the expected values of each element: *E*[*min*(*X*_1_, *X*_2_)] ≤ *min*[*E*(*X*_1_), *E*(*X*_2_)]; see Jensen's inequality (as e.g., described in Rudin, 2006). Owing to this statistical fact, race models are also referred to as “statistical-facilitation” accounts (Raab, 1962). Importantly, on RSTs, no integration or cross-talk is assumed to take place across the two target channels.

Do race models provide a universal account of all RSEs observed empirically? To answer this question, Miller (1982) introduced a bound that formalizes the maximum amount of RSE that a race model can explain: the so-called “race model inequality” (RMI). The RMI relates the distribution function of the redundant-signal reaction times *F*_12_ to the distribution functions of the single-signal reaction times *F*_1_, *F*_2_ (where the indices 1, 2, and 12, denote, e.g., single auditory, single visual, and redundant audio-visual reaction times) given a race model:

(1) $$F_{12}\left(t\right)\leq F_{1}\left(t\right)+F_{2}\left(t\right)\text{​},\text{for all }t$$

Thus, the fastest response times for RSTs can, at the most, be equal to the fastest response time for SSTs. If there are redundant-signal response times that are even shorter, the architecture of race models is not fit to explain the RSE. Thus, the RMI marks a critical test for all race models: any data violating this inequality (at any time point *t*) by definition falsifies of the whole class of race models. Ever since its conception, the RMI was found to be violated in many empirical situations (e.g., Miller, 1982; Grice et al., 1984; Egeth and Mordkoff, 1991; Diederich, 1992; Mordkoff et al., 1996; Krummenacher et al., 2001, 2002; Feintuch and Cohen, 2002; Mordkoff and Danek, 2011; Krummenacher and Müller, 2014).

If the RMI is found to be violated, what architecture then would be responsible for the RSE? Several cognitive architectures have been proposed that can in principle produce RSEs and violations of the RMI: interactive-race models (Mordkoff and Yantis, 1991), serial exhaustive models (Townsend and Nozawa, 1995), correlated-noise models (Otto and Mamassian, 2012), and co-activation models. Of these, co-activation models have mostly been defended successfully against potential alternatives (e.g., Mordkoff and Miller, 1993; Patching and Quinlan, 2002; Zehetleitner et al., 2009).

One possibility, which has only rarely been discussed as a potential cause of RMI violations, is a speed-up of the non-decision components of task performance—rather than of the decision component, as standardly assumed by the accounts mentioned above.

Observed response times may be conceived of as consisting of two components: a decision and a non-decision component (Sanders, 1980; Luce, 1991); in terms of processing stages: perceptual latency, then decision latency, then motor latency, where both the perceptual and motor latencies are combined into a single non-decision component. Consequently, processes responsible for RMI violations can logically stem from either or both of these components. The decision stage is defined as the time needed for a decision variable (e.g., sensory evidence) to trigger a decision required by the experimental paradigm, such as whether a target is present or absent, whether a target is located on the left or the right side of perceptual space, etc. The non-decision time is the sum of sub-processes including stimulus encoding, response selection, and response execution. That is, the non-decision component actually comprises two processing stages: one pre- and one post-decisional. For the sake of brevity, we henceforth use the term non-decision processing stage to summarize both pre- and post-decisional processing. Thus, RMI violations could also be produced by a shortening of the non-decision component on RSTs, compared to SSTs. Such a shortening would result in a shift of the reaction time distribution to the left on the time axis (if the variance of the motor component were left unchanged), thus producing RMI violations. There would be, in principle, other ways of generating RMI violations by the non-decision time alone (though explicated models are lacking in the literature). And, in fact, several scientists have advocated a non-decision locus of RMI violations (see, e.g., Corballis, 1998; Feintuch and Cohen, 2002; Iacoboni and Zaidel, 2003; Miller, 2007; Miller et al., 2009; for a review, see Reynolds and Miller, 2009).

Can one distinguish decisional from non-decisional origins of RMI violations? In order to do so, we used sequential-sampling decision models to account for reaction time distributions in two bimodal RSPs. Sequential-sampling models are based on the assumption that the neuronal states engendered by external stimuli are intrinsically noisy. Such noisy states are sequentially sampled and integrated into sensory evidence until a decision criterion is reached. In the models used here, sensory evidence consists of the accumulated information from sequential samples. The higher the quality of the presented stimulus, the faster this accumulation process reaches the decision criterion (i.e., its drift rate is higher), thus producing faster and more narrowly distributed reaction times, coupled with lower error rates. Additionally, the decision criterion can be low (corresponding to a liberal response criterion), which would give rise to faster and less accurate responses compared to those based on a high criterion. Finally, perceptual and motor latencies are combined into a non-decision time, which has its own distribution (see Section Validity of Model Parameters for empirical evidence that a cognitive interpretation of the model parameters is justified). The observed reaction time distribution is then the convolution of the decision and non-decision time distributions.

In this framework, co-activation models assume that the drift rate on RSTs is higher than the highest drift rate on SSTs. By contrast, a non-decisional origin of the RSE would be reflected in a faster non-decision time parameter for redundant-signals compared to SSTs.—Alterative architectures are considered in the General Discussion.

To date, to our knowledge, only decisional variants of co-activation accounts have been implemented in the form of sequential-sampling models, with the models of Diederich (1995) and Schwarz (1994) both assuming a summation in the rate of evidence accumulation for RSTs over SSTs (see also Blurton et al., 2014). However, there are no studies that attempted to fit non-decisional or combined co-activation accounts (where both decision and non-decision parameters may vary) in a comparative fashion. It is, thus, unclear whether a combined (decision and non-decision) model could outperform a purely decision-based model and how substantial the contribution of a non-decision time shortening might be.

Accordingly, the present study was meant to contribute to the debate on the source of RMI violations, both conceptually and methodologically. In detail, a sequential-sampling model analysis was performed to fit quantile proportions of the response time distributions observed in two bimodal—audio-visual—RSP experiments to three model variants that assume different sources of co-activation: (a) a decisional model (where drift rates may vary), (b) a non-decisional model (where non-decision times may vary), and (c) a combined model (where both drift rates and non-decision times may vary). This way, the question of the origin (s) of RMI violations (and of the RSE in consequence) can be addressed: does co-activation occur at a decisional stage, a non-decisional, or at both stages and, if the latter, to what comparative degree?

On a methodological level, the present study was intended to highlight the applicability of sequential-sampling models to account for reaction time distributions (rather than solely for mean reaction times and their variance) in the RSP, to reveal latent psychological variables and so shed light on the nature of the RSE.

The General Discussion will address aspects of the generalizability of both the general modeling approach and the specific modeling results of the present study, alternative architectures, as well as the notion of the RSE as a theoretical “umbrella term.”

## Materials and methods^1^

In Experiment 1, participants performed a simple reaction time (SRT) task, in which they had to make the same response—simultaneously pressing the two buttons of a standard Microsoft mouse—to the onset of a visual target alone (SST 1), an auditory target alone (SST 2), or an audio-visual target pair (RST). A variable inter-trial interval (ITI) was used to prevent anticipatory or rhythmic responses. In Experiment 2, a two-choice reaction time task was introduced, in which participants were presented with the same stimuli as Experiment 1, which could however appear on the left or the right of perceptual space (i.e., to the left or the right of the fixation cross). Participants' task was to make a speeded two-alternative choice response—by pressing one or the other mouse button—to the side of the target (pair) on a given trial. In all other respects, Experiment 2 was identical to Experiment 1.

### Participants

In *Experiment 1*, 15 participants (11 of them female) performed a single, 45-min session in return for €6.00 or a course credit. Their average age was 25.7 (range: 20–34) years, and they were all right-handed and had normal or corrected-to-normal vision. In *Experiment 2*, 21 new participants (14 of them female) completed a single, 60-min session in return for €8.00 or a course credit. Their average age was 27.2 (range: 18–46) years; one participant was left-handed, and all had normal or corrected-to-normal vision.

### Apparatus and stimuli

The experiments were conducted in a sound-insulated booth, and were controlled by programs using MATLAB (R2009bSP1, Natick, Massachusetts: The MathWorks Inc., 2010) and the PsychToolbox (Brainard, 1997; Pelli, 1997), running on an Apple Mac mini (Cupertino, California: Apple Inc.) computer (with Mac OS X).

The visual stimuli—gray discs (CIE Yxy 10.9, 0.286, 0.333), 1° of visual angle in diameter—were presented on a 20″ Mitsubishi Diamond Pro 2070SB monitor set at a resolution of 1280 × 1024 pixels and a refresh rate of 100 Hz, with a viewing distance of approximately 75 cm. The auditory stimuli were 400-Hz beeps (of a duration of 150 ms) delivered via headphones and redundant stimuli were the combined visual and auditory stimuli, presented simultaneously (i.e., with an onset asynchrony of 0 ms). In Experiment 1, the visual stimuli were presented centrally and the auditory stimuli binaurally, and participants responded to the onset of the respective target stimulus, or pair of stimuli, by simultaneously pressing both (i.e., the left and the right) mouse buttons using their left- and right-hand index fingers (simple reaction time task). In Experiment 2, the stimuli were presented lateralized, and participants responded with the right button to any stimulus, or pair of stimuli, on the right, and with the left button to any stimulus, or pair of stimuli, on the left (left-right forced-choice discrimination task). On RSTs in Experiment 2, the visual and auditory stimuli were always presented on the same side (i.e., either both on the left or both on the right), so that there was never any spatial conflict between the redundant-target signals.

All analyses and the numerical parameter fitting were carried out using GNU R (version 2.14.0). For the fitting procedures, the “optim” package was used.

### Procedure

**Figure 1:**
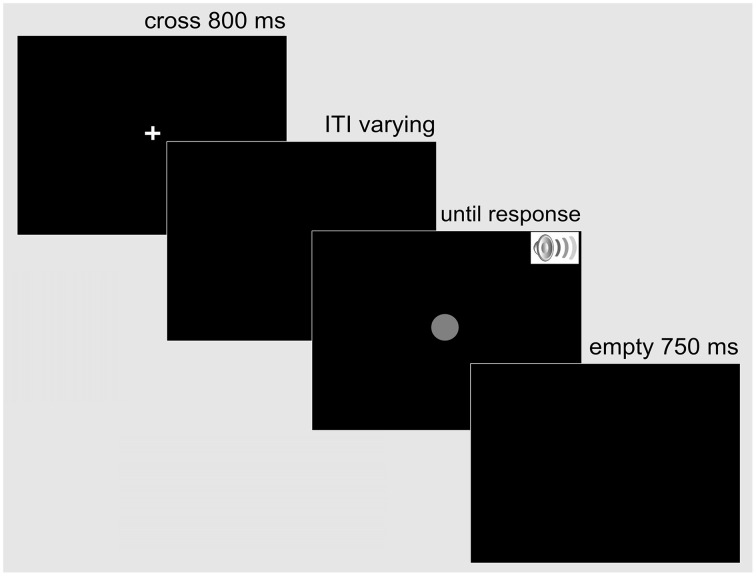
**Example display sequence on a trial in the simple RT Experiment 1**. A trial started with a fixation cross presented centrally for 800 ms. Following a variable inter-trial interval, the response-relevant target—a single auditory (SST auditory), a single visual (SST visual), or a redundant audio-visual stimulus (RST audio-visual) appeared. The auditory stimulus was terminated after 150 ms, while the visual stimulus remained on the screen until the observer responded bimanually. A blank screen followed for 750 ms before the next trial began.

Each trial was structured in the following way: First, a white fixation cross (0.5° × 0.5° of visual angle) was presented centrally on a black screen for 800 ms. Then, after an inter-trial interval (ITI) that varied uniformly between 500 and 1500 ms, the target stimulus or pair of stimuli appeared. The auditory stimulus was terminated after 150 ms, while the visual stimulus remained on the screen until the observer initiated a response. The response was followed by a 750-ms waiting period, after which the next trial started with the fixation cross (see Figure 1 for the sequence of displays on a trial).

Experiment 1 was divided into 17 blocks of 45 trials, with unimodal trials (SSTs) and bimodal trials (RSTs) interchanging randomly. Overall, this amounted to 765 trials (255 trials for each condition, i.e., SST visual, SST auditory, and RST audio-visual). Experiment 2 was divided into 20 blocks of 45 trials, yielding 900 trials in total (150 trials for each condition and screen side, i.e., SST visual left, SST visual right, SST auditory left, SST auditory right, RST audio-visual left, and RST audio-visual right). Participants could take a break in between blocks, and they were provided with feedback about their block mean reaction time and error rate. They were instructed to respond as fast as possible while keeping their error rate below 5%.

As pointed out by Mordkoff and Yantis (1991), violations of the RMI are difficult to attribute to a co-activation model if the experimental design involves contingencies that could benefit redundant-signals over SSTs. Specifically, there are two types of contingencies, inter-stimulus and non-target response benefits. The inter-stimulus response benefit is calculated as Pr(T_A_|T_V_) - *Pr*(T_A_|N_V_), that is, it indicates by how much the conditional probability of an auditory target given that the visual channel detected a visual target exceeds the conditional probability of an auditory target given that the visual channel determined the absence of a visual target. The non-target response bias for redundant targets is calculated as Pr(+) − Pr(+|*N_A/V_*), that is, it indicates by how much the probability of a target (denoted as “+”) exceeds the conditional probability of a target given that no target has been detected in one (the auditory or the visual) channel.

In Experiment 1, the inter-stimulus response benefit was −0.5, Pr(T_A_|T_V_) − Pr(T_A_|N_V_) = 0.5–1, and thus, although present, it worked against redundant-target and in favor of single-target trials. Further, the non-target response benefit was 0, Pr(+) − Pr(+|N_A/V_) = 1–1. However, given that a SRT paradigm was used in Experiment 1, the target could appear in the time interval between 1300 and 2300 ms after the onset of the fixation cross (at the start of the trial). If one divides this 1000 ms interval into two time windows of 500 ms each, both types of contingencies would be benefitting redundant-signals trials, to the numerical value of 0.25 each. In Experiment 2, the two types of contingency benefit were Pr(left_A_|left_V_) - *Pr*(left_A_|right_V_) = 0.5 and Pr(“left”) − Pr(“left”|right_A/V_) = 0.5, respectively.

### Models and fitting

#### Single-boundary accumulation and ratcliff diffusion models

The three co-activation models (the decisional, the non-decisional, and the combined model) were each implemented assuming a noisy accumulation of evidence against one boundary for the SRT experiment, and the Ratcliff Diffusion Model (Ratcliff, 1978) for the two-choice RT experiment. The accumulation of a stochastic source of evidence against one boundary produces a distribution of response times captured by the ex-Wald distribution (Schwarz, 2001). Here, the Wald component is responsible for the distribution of decision times, and an exponential distribution accounts for the non-decision times, which summarize all processes following (and possibly preceding) the decision stage. The parameters of the ex-Wald model are the mean drift rate of accumulation *v*, the decision criterion *a*, and the exponential rate parameter γ = 1/*t*. While single-boundary accumulation models can account for SRT performance, two-alternative choice performance is more appropriately captured by a diffusion process against two decision boundaries reflecting the two response alternatives, such as the Ratcliff Diffusion Model (RDM). The RDM involves seven parameters, the four most important being the drift rate *v*, the criterion *a*, the starting point *z*, and the non-decision time *Ter*. The RDM parameter *z*, controlling the starting point of the evidence accumulation process, was set here to *a/2* for each model (for purposes of simplification), resulting in unbiased evidence accumulation. The variability of the non-decision time *Ter*, *s_t_*, controls the amount of variance of the non-decision component. The parameters η and *s_z_*, the variability of the drift rate and starting point, respectively, were both set to zero (the EZ-diffusion model of Wagenmakers et al., 2007, makes the same simplifying assumptions).

For the decisional model, the respective drift rate parameter ν was free to vary between the two SSTs and RSTs as they control the rate of evidence accumulation over time and thus represent the clarity or “ease of processing” of the signals. For the non-decisional model, the parameters (*t* and *Ter*) were free to vary, as they quantify the mean non-decision time for each accumulation process. The combined model allowed both the drift rate and the non-decision time to vary across conditions. Additionally, a free model was implemented that allowed every ex-Wald and RDM parameter to vary for each condition. This completely unconstrained model, albeit theoretically implausible, was used to assess the general ability of each model to fit the conditions. Table 1 gives an overview of the free and constrained parameters for each co-activation model variant.

**Table 1:** **Co-activation models with free and constrained parameters, and degrees of freedom**.

**Model**	**Free parameters**	**Constrained parameters**	**Degrees of freedom**
**SIMPLE RT (EX-WALD)**			
Decision	ν	*a, t*	5
Non-decision	*t*	ν, *a*	5
Combined	ν, *t*	*A*	7
Free	ν *a, t*	(none)	9
**TWO-CHOICE RT (RDM)**			
Decision	N	*a, Ter, s_t_*	6
Non-decision	*Ter*	ν, *a, s_t_*	6
Combined	ν, *Ter*	*a, s_t_*	8
Free	ν, *a, Ter, s_t_*	(none)	12

#### Quantile distribution functions

In order to find the model (and the respective parameters) that can best explain the data, fitting of quantile proportions was performed. These were computed by use of quantile probability functions. Quantile probability functions plot response probabilities against quantile response times. The probability of a response for a particular stimulus type determines the position of a point on the X-axis, and the quantile RTs for that stimulus type determine the position on the Y-axis (Ratcliff et al., 2004). Quantile functions give a fuller description of the reaction time data than mean and standard deviation values alone, as the proportion in each quantile bin is visible as well as the spread of the entire distribution. **Figure 3** displays the empirical quantile proportions of Experiments 1 and 2. Vincentizing was used to combine the data of all participants for each condition (Ratcliff, 1979). For estimating, quantile definition 7 of Hyndman's sample quantiles was used (Hyndman and Fan, 1996). Consistent with the mean-variance relation, the fastest condition (here, the bimodal, redundant-target trials) also displayed the narrowest response time range (Wagenmakers et al., 2005).

#### Fitting procedure

The generic fitting procedure for each model involved four computational steps. First, a vector of starting parameters was generated randomly. By design, it consisted of the parameters for each of the three target types (i.e., auditory, visual, and audio-visual). The exact composition of this vector varied depending on the model that was being tested. For example, the decisional model only allowed the drift rates to vary; all other parameters were fixed across the three target types.

Second, for that parameter vector, the model cumulative distribution function was calculated, using an R implementation of the ex-Wald densitiy (Heathcote, 2004) and the “fastdm” code for the density of Ratcliff's diffusion model (Voss and Voss, 2007, 2008) to extract the model quantiles, 0, 0.1, 0.3, 0.5, 0.7, 0.9, 1.0.

Third, the quantile response times of the experimental and model data were used to generate the predicted cumulative probability of a response by that quantile response time. Subtracting the cumulative probabilities for each successive quantile from the next higher quantile gives the proportion of responses between each quantile (ideally this yields 0.1, 0.2, 0.2, 0.2, 0.2, 0.1). The observed and expected proportions were multiplied by the number of observations to produce the expected frequencies (see Quantile Maximal Probability, Heathcote et al., 2002).

Fourth, the model fit quality was quantified and minimized, using a general SIMPLEX minimization routine (Nelder and Mead, 1965, implemented in the “optim” package for R), which adjusts the parameters to find those that yield the minimum score for each model (i.e., iterating through steps 2 and 3). As a cost function, the BIC statistic was used (Schwarz, 1978; Raftery, 1986), which penalizes for the complexity (i.e., the degrees of freedom) of the models:

(2) $$BIC=-2\left[\sum N_{i}p_{i}\ln\left(\pi_{i}\right)\right]+M\ln\left(N\right)$$

Here, *p_i_* and *π_i_* are the proportion of observations in the *i-*th bin for the empirical data and the model prediction, respectively, and *M ln(N)* is the penalizing term related to the number of free parameters *M* and the sample size *N*, that is, the number of observations (see Gomez et al., 2007). *N*_i_ denotes the number of observations per bin, with *N* = ∑ *N_i_*, which was calculated by averaging the number of observations over all participants and conditions. The last bin contains the proportion of errors. Bins 1-6 are the inter-quantile proportions for correct responses (i.e., 0.1, 0.2, 0.2, 0.2, 0.2, 0.1 for the quantiles 0.1, 0.3, 0.5, 0.7, 0.9) multiplied by the proportion of correct responses. Thus, the sum of all bin proportions is 1.

The model with the lowest BIC can be considered that which concurrently maximizes descriptive accuracy (goodness of fit) and parsimony (smallest complexity of description, i.e., fewest necessary parameters). The BIC rests on the assumption that the correct model is among the candidate models tested. For advantages and disadvantages of BIC and alternatives (such as the Akaike Information Criterion, AIC; Akaike, 1978, see for instance Burnham and Anderson (2002) and Kass and Raftery (2012) (cf. Wagenmakers and Farrell, 2004). In order to identify the best out of the set of tested model, the raw BIC values were transformed to BIC weights (Wagenmakers and Farrell, 2004; Jepma et al., 2009). The transformation of BIC values involved three steps: First, for each model *i*, the difference in BIC with respect to the model with the lowest BIC value was computed [i.e., Δ*_i_*(*BIC*)]. Second, the relative likelihood *L* of each model *i* was estimated by means of the following transformation:

(3) $$L\left(M_{i}|data\right)\propto \exp\left[-0.5\cdot \Delta_{i}(BIC)\right]$$

where ∝ stands for “is proportional to.” Third, the model probabilities were computed by normalizing the relative model likelihoods, by dividing each model likelihood by the sum of the likelihoods of all models. The values thus derived for each model are referred to as BIC weights, *w_i_*(*BIC*) for each model *M_i_* and *w_i_*(*BIC*) can be interpreted as the probability that model *M_i_* is correct, given the data, the set of models, and equal priors on the models (Wagenmakers and Farrell, 2004).

#### Model selection

The fitting procedure was performed by randomly sampling initial parameter values (1000 times) and performing the four computational steps described above. This procedure was followed to assure that local minima were avoided in the optimization algorithm. The minimum cost value for each condition was used to assess which model was in best agreement with the data and with which specific parameter vector.

#### RMI analysis

For the analysis of violations of the RMI, we used Ulrich et al. (2007) algorithm for calculating the empirical cumulative density functions. First, for each participant, we calculated the magnitude of RMI violations

(4) $$d\left(t\right)=G_{AV}\left(t\right)-min\left[G_{A}\left(t\right)+G_{V}\left(t\right),1\right],$$

where *G_AV_*, *G_A_*, and *G_V_* stand for the estimates of the empirical cumulative density functions for the redundant, single audio, and single visual trials, respectively (using Ulrich et al.'s, 2007 algorithm; Equation 3). Then, *d*(t) was evaluated at the 0.05, 0.1, …, 0.95 quantile RTs of the redundant trials. For each percentile, *d*(t) was tested against zero, *d*(t) > 0, using using a two-tailed *t*-test, with the alpha level Bonferroni-corrected to 0.0026 (= 0.05/19 probability points).

## Results

### Errors

Errors were defined as anticipatory responses (RT ≤ 150 ms) or misses (RT > 1600 ms). Participants committed 2.00% errors (1.34% anticipations and 0.66% misses) in Experiment 1 and 3.4% in Experiment 2. For each experiment, the data of one participant had to be discarded due to error rates greater than 10 and 20%, respectively.

### Mean reaction times and RSEs

The mean RTs for both experiments are listed in Table 2. Although numerically different, both unimodal conditions in Experiment 1 were statistically the same. There were pronounced RSEs of 55 and 50 ms for Experiments 1 and 2, respectively. The mean RSEs and their standard deviations were computed by calculating the difference of the mean in the RST condition from that of the faster one of the two SST conditions, for each participant.

**Table 2:** **Mean Response Times and RSEs (standard deviations in parentheses) for unimodal (auditory, visual) and bimodal (audio-visual) stimulus conditions in the simple RT Experiment 1 and the two-choice RT Experiment 2**.

**Condition**	**Simple RT**	**Two-choice RT**
Auditory	352 (84)	406 (65)
Visual	383 (74)	409 (63)
Audio-visual	294 (58)	345 (53)
RSE	58	61

### RMI violations

**Figure 2:**
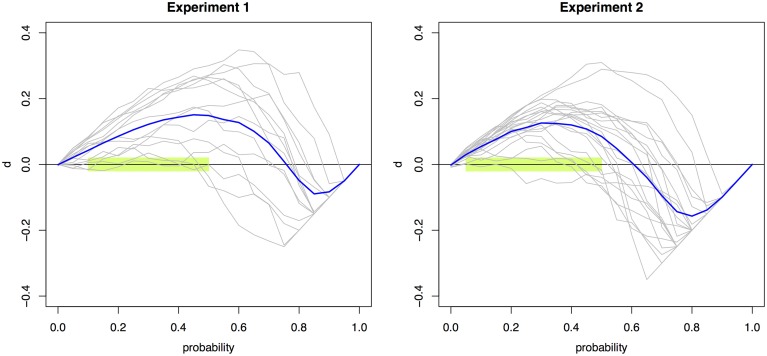
**Violations of the RMI**. The race model test function *d*(*t*) aggregated across individual observers (blue line) and for each individual observer (gray lines) for Experiments 1 (left) and 2 (right). Values that are significantly above zero constitute violations of the RMI. Violations were obtained for the probability points 0.05–0.50 using multiple *t*-tests with a Bonferroni-corrected significance level of 0.0026. This region is highlighted in light green.

Significant violations (*p* < 0.0026) were found across 10 and nine probabilities (0.05 to 0.50 and to 0.45) for Experiments 1 and 2, respectively. Figure 2 presents the individual and mean RMI test function d(t) curves for Experiment 1 and 2 (Colonius and Diederich, 2006). The RMI test function plots the difference between the single-signal distribution and the redundant-signals distribution. Any area above the X-axis signifies violations of the RMI; areas below are in accordance with the RMI bound.

### Fitting results

On the level of mean RTs, all implemented model variants (except the simple-RT decision model) were able to reproduce the reaction time patterns for both experiments. None of the models could generate the standard deviation for every experimental condition; rather, they tended to overestimate the standard deviations. In the simple-RT fitting, the decision model proved unable to produce the empirical RSE; and in the two-alternative choice RT fitting, the non-decision model was unable to fit the RSE. See Table 3 for a list of mean reaction times, standard deviations, and RSEs.

**Table 3:** **Mean response times (and standard deviations in parentheses) of the empirical and model data for Experiment 1 (simple RT) and Experiment 2 (two-choice RT)**.

**Condition**	**Model**				
	**Empirical**	**Decision**	**Non-decision**	**Combined**	**Free**
**SIMPLE RT**					
Auditory	352 (84)	333 (90)	347 (88)	346 (109)	348 (112)
Visual	383 (74)	369 (92)	373 (110)	376 (102)	375 (104)
Audiovisual	294 (58)	303 (89)	281 (51)	285 (62)	283 (61)
RSE	58	30	66	62	61
**TWO-CHOICE RT**					
Auditory	406 (65)	406 (118)	395 (100)	406 (114)	407 (112)
Visual	409 (63)	410 (122)	406 (100)	410 (106)	413 (110)
Audiovisual	345 (53)	346 (68)	360 (100)	345 (80)	343 (79)
RSE	61	61	35	61	64

The outcome of the fitting procedure for Experiments 1 and 2, however, produced a clear separation among the models. Table 4 lists the minimum BIC values for all models, separately for Experiments 1 and 2. For both experiments, the combined model turned out to be best-fitting model. The combined model of the two-choice RT data exhibited an even better fit than the fully unconstrained model, though only because the latter suffered a larger BIC penalty for its extra free parameters. Interestingly, the composition of the RSEs differed between the best-fitting simple-RT and two-alternative choice RT models. In the combined model for the simple-RT data, the non-decision component contributed to 78% of the RSE; in the combined model of the two-alternative choice RT data, by contrast, 58%.

**Table 4:** **Minimum BIC values (and degrees of freedom in parentheses) and BIC weights for each model, separately for the simple RT data (Experiment 1) and the two-alternative choice RT data (Experiment 2)**.

**Model**	**Simple RT**		**Two-choice RT**	
	**BIC (DoF)**	***w(BIC)***	**BIC (DoF)**	***w(BIC)***
Decision	2692 (5)	0.0003	3182 (6)	<0.0001
Non-decision	2713 (5)	<0.0001	3163 (6)	0.1439
Combined	2675 (7)	0.9926	3160 (8)	0.8561
Free	2685 (9)	0.0071	3180 (12)	<0.0001

**Figure 3:**
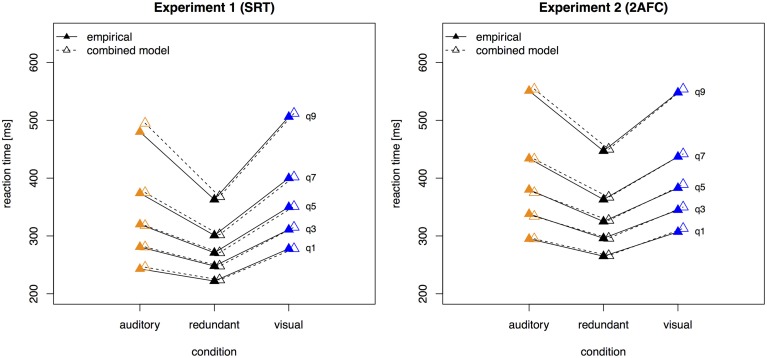
**Quantile reaction times**. Quantile reaction times for the combined model and empirical data from Experiment 1 (left panel) and Experiment 2 (right panel). Continuous lines and filled pyramids denote the empirical data, dashed lines and empty pyramids the model data.

Figure 3 presents the quantile function plots of the combined model for Experiments 1 and 2, respectively.

### Parameter analysis

From a qualitative view, arguably, the free, motor, and combined models agree well with regard to the range of the drift rates, criteria, and non-decision times for the three conditions. All models yielded the highest drift rate parameter and the lowest non-decision time for the redundant condition (where these parameters are allowed to vary). Table 5 gives an overview of the best fitting parameters per model.

**Table 5:** **Parameter values of the fitted models, separately for Experiments 1 (simple RT) and 2 (two-coice RT)**.

**Model**	**Parameter**											
	***v_a_***	***v_v_***	***v_av_***	***a_a_***	***a_v_***	***a_av_***	***t_a_***	***t_v_***	***t_av_***	***st_a_***	***st_v_***	***st_av_***
**SIMPLE RT**												
Combined	21.53	18.59	22.8	5.16	5.16	5.16	0.11	0.10	0.06			
Decision	19.94	17.4	22.64	4.91	4.91	4.91	0.09	0.09	0.09			
Non-decision	10.46	10.46	10.46	2.88	2.88	2.88	0.07	0.1	0.01			
Full	18.17	25.17	21.27	4.37	6.86	4.8	0.11	0.10	0.06			
**TWO-CHOICE RT**												
Combined	3.41	3.62	4.49	1.23	1.23	1.23	0.023	0.025	0.021	0.02	0.02	0.02
Decision	3.42	3.34	5.27	1.26	1.26	1.26	0.23	0.23	0.23	0.02	0.02	0.02
Non-decision	3.79	3.79	3.70	1.23	1.23	1.23	0.24	0.25	0.20	0.02	0.02	0.02
Full	3.59	3.58	4.29	1.28	1.24	1.1	0.23	0.24	0.22	0.07	0.02	0.02

## Discussion

### Observers' performance

The low error rates across the two experiments indicate the general simplicity of the tasks and attest to our observers' ability to follow the instructions. On a mean level analysis, the experiment demonstrated pronounced RSEs of 55 and 50 ms (in Experiments 1 and 2), respectively. Comparing the two single target conditions in Experiment 1, auditory-signal trials were processed faster than visual-signal trials. Albeit not statistically significant, this is in accordance with basic findings (Todd, 1912) of faster response times to auditory than to visual stimuli (for medium intensity levels). In Experiment 2, the two unimodal conditions differed neither numerically nor statistically.

The many RMI violations—obtained for ten quantiles in Experiment 1 and nine in Experiment 2—effectively rule out the class of race models as explanatory accounts for the simple RT and the two-alternative choice RT data. This conclusion is underscored by the facts that both a conservative α-correction was used and response contingencies were avoided (Mordkoff and Miller, 1993). The RMI violations occurred in the lower range of probability points, which is of course plausible given the “make-up” of the RMI. Overall, these results indicate that the empirical RT data cannot be accounted for by a race model architecture.

### Validity of model parameters

In general, the model parameters used here are mathematical constructs that, by mathematical transformations, yield distributions which can be compared to empirical reaction time distributions. The conclusions of the present study are based on the assumption that the different parameters of the decision models indeed map onto cognitive processes—specifically that the drift parameter *v* maps to stimulus quality and the parameters *Ter* and *t* to non-decision times; and that parameter *a* maps to response caution. Here, we review four studies which argue that this mapping is indeed justified.

In all of these studies, experimental manipulations were used to manipulate those cognitive aspects of processing that decision models' parameters are supposed to map onto. Specifically, manipulations comprised stimulus difficulty (Schwarz, 2001; Voss et al., 2004; Philiastides et al., 2014; van Vugt et al., 2014), response caution (Schwarz, 2001; Voss et al., 2004), and duration of response execution (Voss et al., 2004).

Voss et al. (2004) investigated four experimental conditions in a two-alternative color discrimination task, a baseline condition, and three variations. In the first variation, stimulus discriminability was manipulated by making the two possible colors more similar to each other. In the second variation, observers were instructed to perform the task carefully and avoid making mistakes. In the third variation, the response scheme was manipulated: instead of using two different fingers for the two responses, participants were allowed to use only one, single finger to submit one of the two responses. In accordance with the psychological interpretation of model parameters, drift rates were lower for the manipulation of stimulus discriminability, the two response boundaries were separated more widely when observers followed a conservative (error-avoiding) strategy, and the non-decision parameter increased substantially when the motor response required a more time-consuming movement.

For the ex-Wald model, in a “go/no-go” task, Schwarz (2001) used a digit comparison paradigm: observers, on each trial, were presented with one digit; they had to press a button if the number was greater than five, but withhold a response if the digit was less than five. Schwarz manipulated decision difficulty of discrimination and proportion of “go” responses in a crossed design. Supporting the usual psychological interpretation of decision model parameters, difficulty affected the drift parameter and proportion of “go” responses the threshold parameter. Importantly, neither of the two manipulations affected the non-decision parameter.

Recently, diffusion model parameters have been related to electrophysiological markers of the lateralized readiness potential (LRP), a difference wave between centrally located scalp potentials that usually are evoked by manual responses. van Vugt et al. (2014) found a consistent relation between diffusion model parameters with the temporal dynamics and shape of averaged LRPs. Taken together, they found that the ramping up of activity in the LRP is related to the accumulation of evidence toward a threshold. Importantly for the present context, van Vugt et al. used the LRP wave to estimate perceptual and motor latency. They calculated, for each observer, perceptual latency as the time at which the stimulus-locked LRP deviated from baseline activity, and motor latency as the time from the peak of the response-locked LRP to the manual response. The sum of these two latencies thus provided an estimator of non-decision time based on EEG data. This electrophysiologically derived estimator was significantly correlated with the non-decision time parameters individually recovered from a diffusion model fit to the behavioral data.

Finally, Philiastides et al. (2014) also investigated the relation between the parameters of a diffusion model fit to two-alternative choice behavioral data and single-trial EEG traces. First, they found that the model that best captured the behavioral changes induced by a manipulation of stimulus quality only had drift rate as a free parameter. Additionally freeing non-decision time to vary between stimulus conditions did not improve the fit any further. Moreover, of importance in the present context, they extracted, from single-trial EEG, a signal that best differentiated between the low- and high-quality stimulus conditions. The onset time of this extracted signal, that is, the time from stimulus onset until stimulus quality has differential effects on the EEG signal, can be considered as a marker of non-decision processing time. This onset time was found to correlate strongly with individually fitted non-decision time parameters of the diffusion model.

In sum, these studies strongly indicate that the parameters of decision models, especially non-decision time parameters, are indeed related to the corresponding cognitive processes. Thus, arguably, it is justified to interpret our finding of redundant signals to affect non-decision time parameters as reflecting cognitive non-decision processing.

### Decision and non-decision processes contribute to RMI violations

**Figure 4:**
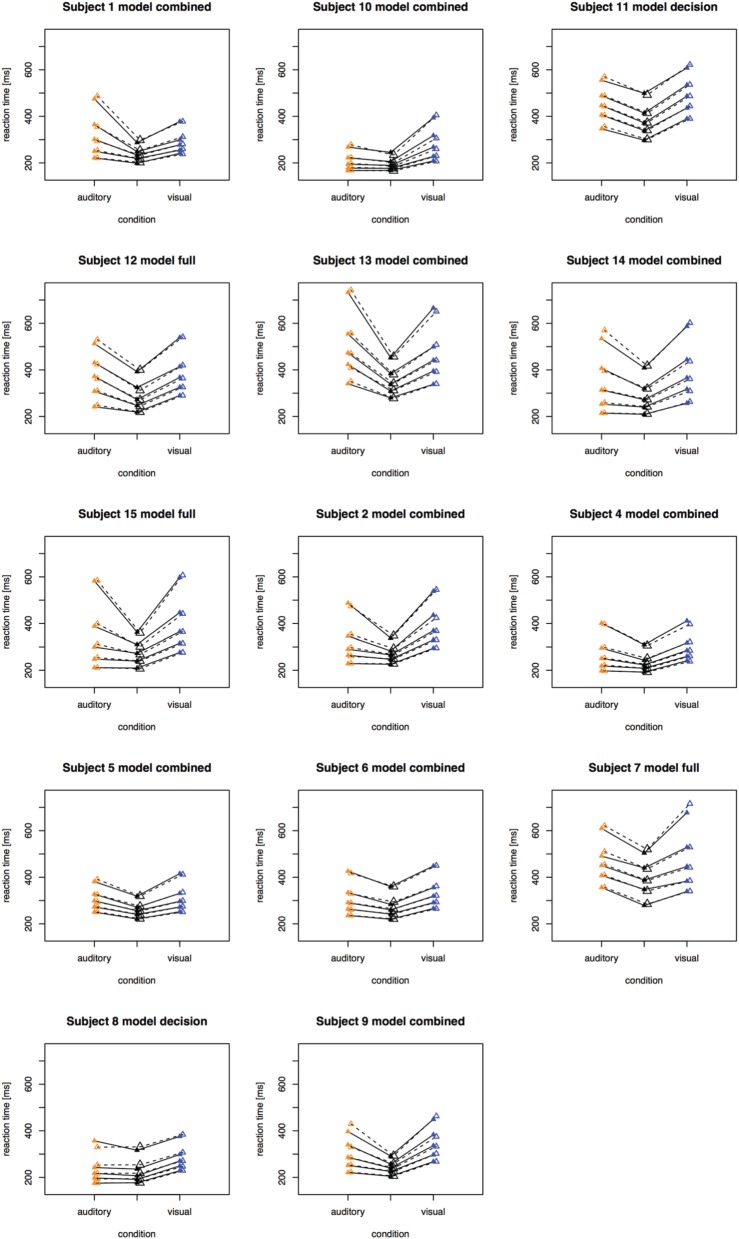
**Quantile reaction times**. Quantile reaction times for single subjects in Experiment 1. The type of best fitting model is indicated in the each figure heading.

**Figure 5:**
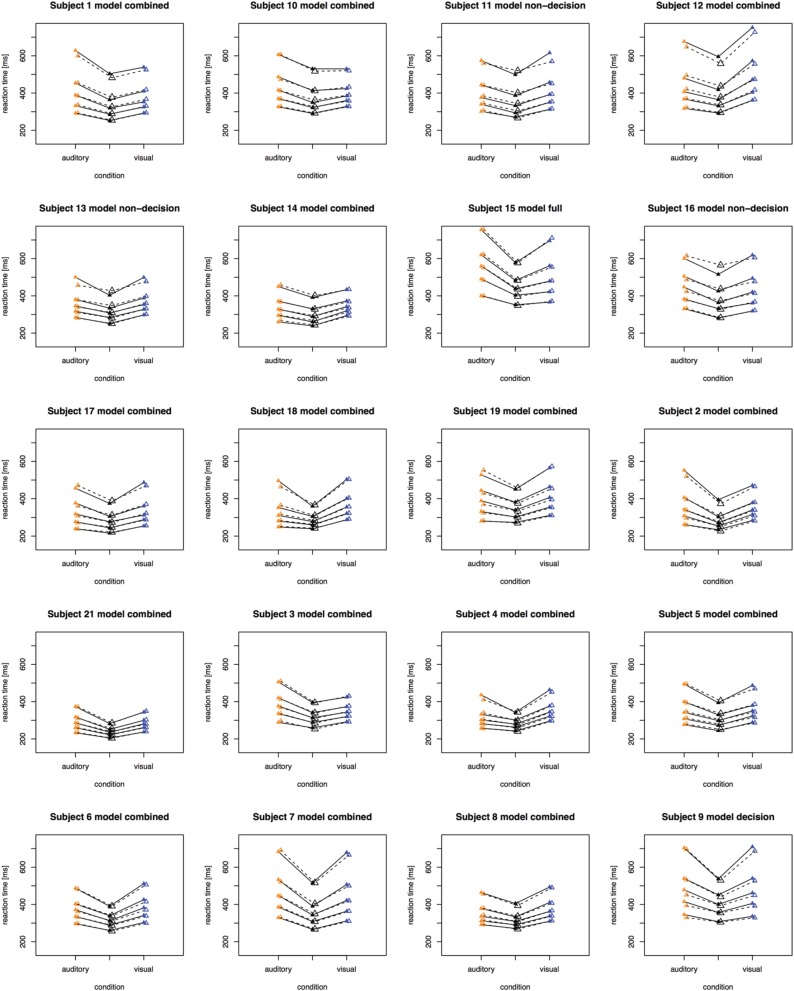
**Quantile reaction times**. Quantile reaction times for single subjects in Experiment 2. The type of best fitting model is indicated in the each figure heading.

The fitting results indicate that the best-fitting account for both the simple RT and the two-alternative choice RT data is provided by the combined model, in which the drift rates and non-decision times are allowed to vary across all conditions. This model is clearly set apart from the next best-fitting model, as the cost function is defined on a logarithmic scale. Inspection of the parameters (of the combined models) revealed that all models yielded a comparable parameter value range, which points to the reliability of the fits. Also, all models shared a pattern across both experiments: for all models, redundant-signals trials exhibited the highest drift rates and the shortest non-decision times. Together with the BIC scores, this can be taken as evidence for a combined drift rate and non-decision component account for the data of the present, bimodal RSP experiments. However, the models were fitted to the average (vincentized) distribution of the whole sample of participants. Thus, it remains possible that some participants actually exhibited purely decisional and others purely non-decisional origins of the RSE and that their mixture is responsible for the best-fitting model being the combined one. To examine this, we also fitted the models to each, single participant's data. In Experiment 1, the decision model, the non-decision model, the combined model, and the full model provided the best fit for 2, 0, 10, and three participants, respectively. For Experiment 2, the best fitting models were one times the decision model, three times the non-decision time model, 16 times the combined model, and one times the full model. That is, even for model fits on the level of single participants, the combined model provided the best fit for the large majority of the participants (see Figures 4, 5 for individual results).

Given that the fitting results do indeed reveal the generating mechanisms for the data obtained in the two experiments, the decisional and non-decisional components would appear to be contributing differentially to the total, observed RSEs. In Experiment 1, of a total RSE of 56, 43 ms are attributable to the non-decision time difference between the faster of the two unimodal conditions and the bimodal condition alone. In contrast, in Experiment 2, just half the RSE—26 ms of a total 51 ms—can be attributed to this non-decision time difference. This outcome would be consistent with Miller (1982), who hinted at the possibility of the RSE being a mixture of both decisional and non-decisional processes.

Studies that have tried to fit data to explicit co-activation models are rare. One of the explicit models, which assumes co-activation at the decisional stage, is Schwarz's (2001) superposition model. The basic assumption of Schwarz's model is that, on redundant-target trials, the separate activations of the two stimulated channels superpose to form the overall-diffusion process, where sensory evidence on RSTs is the sum of sensory evidence from the two single channels: *X*_12_(*t*) = *X*_1_(*t*) + *X*_2_(*t*). Activity in both channels can be adequately described by independent diffusion processes of the Wiener type and can have variable channel dependency. Applying Schwarz's superposition model to data from Miller (1986) achieved a good prediction on the level of mean reaction times.

Diederich (1995) conducted a trimodal simple-RT study with visual, auditory, and tactile stimuli, with varying inter-stimulus intervals, and fitted a race model and two co-activation models to empirically observed RT means and variances. Although both co-activation models outperformed the separate-activations model and yielded excellent fits of the mean reaction time, Diederich notes that they failed to adequately capture the spread of the response times.

In line with the present diffusion model analysis, Diederich and Colonius's (1987) study also yielded positive evidence for co-activation occurring at the non-decision stage: examining the distributions of RT differences between left- and right-hand responses revealed a U-shaped dependence of the amount of facilitation in the motor component on the inter-stimulus interval. Note though that this analysis based on RT differences rests upon the (disputable) assumption that the motor delay constitutes an additive component of the entire observable RT (see, e.g., McClelland, 1979).

However, a comparison with the studies of Diederich (1995) and Diederich and Colonius (1987) remains problematic. Both studies examined the goodness-of-fit only for decisional models and only at the level of reaction time means and variances—rather than the complete reaction time distribution (see also Blurton et al., 2014). In the present study, relying on the fit to the means alone would not have helped distinguish between the decisional and combined models in Experiment 1. And for Experiment 2, such an analysis would not have allowed us to rule out any of the models. As the decisional model involved the lowest number of parameters (namely, six) compared to the other models, the principle of parsimony would imply a preference for decisional models—though even for Experiment 2, the decisional model exhibited the poorest fit. On a methodological level, these differential outcomes provide a strong argument in favor of the use of distributional analyses of sequential-sampling models and against fitting decision models only to reaction time means and variances.

However, it must be acknowledged that the data from these two tasks were analyzed using different models (ex-Wald vs. RDM), so that the difference in RSE sources observed might be attributable, at least in part, to the difference in the models, rather than the tasks, employed. Specifically, in the RDM, the non-decision component has a uniform distribution, whereas in the ex-Wald model, the non-decision time has an exponential distribution. Perhaps the exponential rather than uniform non-decision component is responsible for non-decision time to exhibit a larger contribution to the RSE than the decision component^2^. In order to examine this possibility, we fit a RDM model to the SRT data from Experiment 1, and an ex-Wald model to the 2AFC data from Experiment 2. To do so, in the RDM, we set the separation from the starting point to the negative response boundary at a very high value, so as not to produce decision errors. Apart from that, the fitting routines and data were the same as above. Both for the data of Experiment 1 and for the data from Experiment 2, the best fitting model with the lowest BIC was the combined model, where target redundancy affected both the drift rate and the non-decision time (as compared to the pure drift and the pure non-decision time component), thus replicating the model ordering of the original fitting. Furthermore, for the data of Experiment 1 fit with a RDM, and for the data from Experiment 2 fit with the ex-Wald model, 4% (Experiment 1) and, respectively, 57% (Experiment 2) of the RSE was attributable to non-decision time—which compares with 78% (Experiment 1) and 58% (Experiment 2) in our original fit. It has to be noted, though, that the RDM model fit to the data of Experiment 1 yielded near-zero variance (ca. 4 ms) of the non-decision component, which is likely indicative of an overestimation of the variance of the decision component. Given that the ex-Wald model explicitly describes the decision mechanism of a go/no-go task and the RMD model that of a 2AFC task, these “cross-task fitting” results must be viewed with caution. For the data of Experiment 2, the proportion of the RSE attributable to the non-decision component was equivalent whether it was fit with a RDM or an ex-Wald model; by contrast, this proportion changed for the data of Experiment 2. Nevertheless, for the cross-fitting too, the best model out of the set of candidates was the combined model. Whether and to what degree the contributions of the decision and non-decision components to the RSE differ between tasks cannot be decided on the basis of the present results.

### Generalizability

We showed that both our experiments yielded RSEs that cannot be accounted for by race model architectures. There are, however, other accounts that can, in principle, produce the critical RMI violations. However, the question of whether these alternatives would involve a non-decision component is fundamental and pertinent to all of these models. Interactive-race models (Mordkoff and Yantis, 1991) are similar to race models but allow for cross-talk between the two single-signal channels: when one channel registers activity, this can lead to a reduction of the drift rate in the other channel. Another model that could account for RMI violations is the serial exhaustive model (Townsend and Nozawa, 1997), according to which, as the name implies, both feature channels (e.g., visual, auditory) are processed in series and exhaustively. This model can generate RMI violations provided that the non-target channel accumulates evidence at a slower rate than the target channel. Another, conceptually different cause of RMI violations would be the presence of response contingencies (Mordkoff and Yantis, 1991; Mordkoff and Miller, 1993). As our study design included such contingencies (see Section Procedure above), we cannot firmly rule out response contingencies as an additional source of the RMI violations. However, as there are currently no explicit generative formulations of these alternative accounts, they cannot, at present, be assessed against the empirical data. Note, though, that the framework of our fitting procedure allows for extensions and adaptations that would make such a model comparison feasible in principle.

In order to corroborate our fitting results and validate the identification of decision and non-decision components in the reaction time data, we additionally performed a validation fitting with synthetically produced RSEs. To this end, we generated three sets of reaction time data (using the ex-Wald and RDM models). One set featured a purely decision-based RSE, generated by models in which only the decision parameter differed between SSTs and RSTs. Another set of data featured a purely non-decision based RSE, generated by analogously changing only the non-decision time parameter across SSTs and RSTs. Lastly, a combined decision/non-decision-based RSE was built into a data set. These three sets of data were then subjected to fitting to all three model types examined (see Section Single-boundary Accumulation and Ratcliff Diffusion Models and Table 1 above). The fitting results showed that all built-in RSEs could be recovered and correctly identified by the fitting procedure, that is: the decision based RSE was best fitted by the decision-only model, and so on. Although the parameter values were not recovered numerically, the qualitative pattern was the same, in terms of the order of the fits and parameter relations. This validation fitting strengthens the results of the fitting of the empirical reaction time data and serves as a proof of concept: it is possible and meaningful to investigate the decision and non-decision components of the RSE employing (generative) reaction time models and fittings on the distributional level. Note that this validation procedure was based on the assumption that the data were indeed generated by the exact model that was used to fit the data. To our knowledge, it is an open question what the implications would be with regard to the validity of a model fit if the empirical data were generated by a mechanism that is different to that assumed by the model used to fit the data.

### The redundant signals effect—an umbrella term?

Other studies that used different experimental paradigms (stimuli, tasks, modalities) have focused solely on a decisional origin of the RSE. The present results however raise the fundamental question whether “RSE” is, in fact, an umbrella term for different phenomena which share the general property of “multiple evidence sources” for performing a perceptual-motor task. Similar notions have already been put forward by Reynolds and Miller (2009) as well as (Schulte et al., 2006). It is, thus, likely that for specific stimulus properties (luminance, spatial frequency, orientation, etc.), tasks of differential complexity (detection, go/no-go, discrimination, etc.), uni- vs. multi-modal paradigms, the RSE is in fact generated by a combination of different mechanisms—and thus to be appropriately accounted for by different types of models. Similarly, Corballis (2002) showed that the RSE is subject to a substantial amount of inter-individual variability. Accordingly, inferences and generalizations across the many variations of the RSE paradigm, and perhaps even across participants, would appear problematic if the data basis is heterogeneous, gathered under very different experimental conditions. In this situation, a sequential-sampling model analysis can help systematize potential sources of the RSE across different paradigm variations and settings.

In summary, the present study examined the locus or loci of the RSE by applying a sequential-sampling model analysis to two bimodal, target detection and left-right localization, tasks. The fitting results challenge the view that co-activation in the RSP is a purely decisional effect. This pattern was even more pronounced in the data of Experiment 2, where the decisional model fared worst and the purely non-decisional model turned out second best in goodness-of-fit terms. Although two experiments are clearly insufficient to definitely rule out a decision-only model, their results emphasize the role of the non-decision stage as a potential source of co-activation effects. Moreover, the results illustrate the usefulness of a systematic sequential-sampling model analysis for situations where the RMI is violated.

Thus, in conclusion, in order to achieve a realistic picture of what the sources of the RSE actually are and how the RSE is composed, a comprehensive series of experiments would be required that elaborate exactly what roles, in the RSP, are played by the stimuli, sensory modalities, response effectors, and experimental tasks in producing co-activation effects and exactly what the generating mechanisms are.

### Conflict of interest statement

The authors declare that the research was conducted in the absence of any commercial or financial relationships that could be construed as a potential conflict of interest.

## Footnotes

^1^The raw data, the analysis codes, all model codes, and the reported results are publically available at the Open Science Framework (osf.io/7hbj6), to facilitate reproduction of the present study and replication of its results (for the open-data and open-code idea, see, e.g., Ince et al., 2012; Morin et al., 2012; Wicherts and Bakker, 2012; Simonsohn, 2013; Wicherts, 2013).

^2^We are grateful to one of the reviewers for pointing out this possibility.
